# Effect of Surface Activation on the Microstructure and Corrosion Resistance of MAO/Ni-P Composite Coating on AZ91D Magnesium Alloy

**DOI:** 10.3390/ma16186185

**Published:** 2023-09-13

**Authors:** Qi Xu, Peng Zhou, Tao Zhang, Fuhui Wang

**Affiliations:** Shenyang National Laboratory for Materials Science, Northeastern University, 3-11 Wenhua Road, Shenyang 110819, China; 2010234@stu.neu.edu.cn (Q.X.); zhoupeng@mail.neu.edu.cn (P.Z.); fhwang@mail.neu.edu.cn (F.W.)

**Keywords:** electroless coating, pretreatment, activation, EIS, corrosion resistance

## Abstract

The purpose of this study is to improve the number and distribution of active particles on the MAO layer by changing the activation method, thus improving the corrosion resistance of the coating. The structure of the coatings was characterized by SEM, XRD, XPS, and AFM, as well as the corrosion resistance of the coatings by polarization curves, EIS tests, immersion tests, and salt spray tests. The conductive resistance and adhesion of different composite coatings were compared. The results demonstrate that the properties of the composite coating are significantly affected by different activation methods, and the Ni-P coating prepared with more active particles offers superior corrosion protection to the inner layer. The quantity and distribution of active particles affect the compactness of the coating by influencing the initial deposition process. The size of nickel particles is larger and the inter-grain porosity increases in the case of fewer active sites, and as the number of active sites increases, the size of nickel particles decreases, and the coating compactness increases. The mechanism of the effect of the number of active particles on the deposition process of electroless Ni-P coating was proposed.

## 1. Introduction

As the demand for lightweight alloys has increased rapidly in advanced manufacturing industries due to environmental concerns and technological advances, over the past decade, magnesium alloy has become one of the most promising structural materials [1,2,3,4]. However, the relatively low hardness and wear resistance, especially the electrochemical activity, limits its wide applications [5,6,7,8]. The need to improve the corrosion resistance of magnesium alloys has inspired the development of protective coating technologies, such as anodic oxidation [9,10], conversion coatings [11,12], vapor deposition [13,14], and thermal spraying [15,16]. These surface treatment technologies offer significant advantages for the development of magnesium alloy applications [17].

With the widespread use of magnesium alloys in aerospace, wear resistance, electrical conductivity, and corrosion resistance are essential requirements for coatings. However, the application of surface treatment technologies for magnesium alloys is limited by some drawbacks. MAO coatings provide excellent hardness and wear resistance, but without electrical conductivity [18,19,20]; the chemical conversion coating exhibits poor wear resistance [12]. Electroless nickel plating produces coatings that possess high wear resistance, electrical conductivity, and corrosion resistance, and thus could be a preferred solution in commercial production [21]. However, the nickel coating on magnesium alloys poses significant problems, as the excessive potential difference between the substrate and the coating can lead to serious galvanic corrosion. A growing interest has been placed in the use of hybrid processes to create middle layers to prevent this problem. Huo [22] and Song et al. [23] used a chemical conversion coating or MAO coating as a middle layer to reduce the potential difference between the substrate and the nickel coating so as to reduce the risk of galvanic corrosion and the rough porous structure of the conversion coating, making so that the MAO layer can provide better adhesion.

However, a substrate that exhibits catalytic activity would be necessary for the deposition process of the electroless plating, a characteristic that MAO coatings do not possess [24,25]. Therefore, the key challenge in electroless plating of MAO layers is the need for a catalytic surface to initiate deposition. V. Ezhilselvi et al. employed NaBH_4_ to reduce Ni^2+^ during the activation process to provide nucleation sites for the deposition of Ni-P coatings on MAO layers [26].

Various processes have been developed to achieve selectivity in the electroless plating of inert surfaces. Sun et al. applied an epoxy film layer containing TiB_2_ powders on the surface of anodized magnesium alloy to obtain catalytically active surfaces [27]. Song and coworkers employed silver ion inks as the activating agent for the electroless plating of MAO coatings [23]. Zhang et al. immersed ceramics in a solution containing nickel sulfate and sodium hypophosphite, and the deposition was triggered by the addition of trace amounts of sodium borohydride, which resulted in the formation of a large number of spherical active nanoscale Ni-P particles on the ceramic’s surface [28]. However, most studies on activation are aimed at making inert surfaces catalytically active. The formation of active sites on the surface of the MAO coating where adsorption and nucleation can take place is the purpose of activation. In contrast, studies on whether the number and distribution of active sites will have an effect on the Ni coating are still lacking.

The study aims to improve the number and distribution of active particles on the MAO layer by changing the activation method, thus improving the corrosion resistance of the coating. The surface and cross-sectional morphology, phase composition, conductive resistance, adhesion, and corrosion resistance of coatings obtained by different activation methods were characterized. The effect of different numbers and the distribution of active particles on the initial deposition process was investigated. The mechanism of the influence of different numbers of active particles on the coating deposition process was proposed.

## 2. Experimental

### 2.1. Materials

The AZ91D magnesium alloy was used as the substrate. The specific composition of the alloy is as follows: Al 8.96 wt. %; Zn 0.64 wt. %; Mn 0.16 wt. %; Si 0.01 wt. %; Fe ≤ 0.005 wt. %; Ni ≤ 0.005 wt. %; Cu ≤ 0.001 wt. %; Ca ≤ 0.001 wt. %; Zr ≤ 0.001 wt. %; and Mg balance. The substrates were cut into 20 mm × 20 mm × 5 mm pieces before being ground sequentially with 400, 600, and 1000 grit silicon carbide sandpaper. The substrates were all cleaned ultrasonically in anhydrous ethanol before being dried and then prepared for composite coating.

### 2.2. Preparation of Composite Coatings

**Figure 1:**
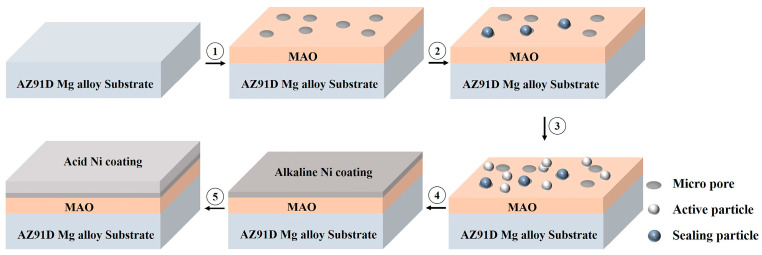
Process of Composite Coating Preparation. (1) MAO process of AZ91D; (2) Sealing; (3) Activation; (4) Alkaline electroless nickel plating; (5) Acid electroless nickel plating.

The preparation process of the composite coating is shown in Figure 1. The preparation of the composite coating consists of five main steps: (1) The MAO coating of the magnesium alloy was prepared in pulse mode. (2) The samples were immersed in the chemical solution for sealing. (3) In order to obtain different numbers and distributions of activated particles, various concentrations of silver nitrate were used for sensitization, followed by activation under different methods. The specific activation methods are listed in Table 1. (4) The activated samples were immersed in an alkaline bath for pre-plating. (5) The pre-plated samples were immersed in the acidic bath. The final composite coating obtained after five steps was recorded as N2-EN, N10-EN, and US10-EN, respectively. Specific experimental parameters and compositions are listed in Table 2.

### 2.3. Microstructural Characterization

The microstructure of the sample was observed via scanning electron microscope (SEM, XL-30 FEG, Philips, Amsterdam, The Netherlands), and the samples were sprayed with gold before observation. The elemental distribution was analyzed with the energy dispersive X-ray spectroscope (EDS) affiliated with SEM. An Xpert Pro X-ray diffractometer (XRD) was used to analyze the phase composition of the different coatings. X-ray photoelectron spectroscopy (XPS) was employed to analyze the XPS spectra and valence band information of the samples with an excitation source of Al-Ka (1486.6 eV). The operating parameters were 1 μA and 1 kV. Roughness analysis of coated surfaces was performed by Atomic Force Microscope (AFM).

### 2.4. Conductive Resistance and Adhesion Tests

The conductive resistance of coatings was tested by ROOKO Surface Resistance Tester (FT-400AHXM, Ningbo, China) according to MIL-DTL-81706B. For adhesion tests, which were performed with automatic adhesion tester (AT-A, DeFelsko, New York, NY, USA) according to ASTM D4541-09. The tensile test column was stuck to the surface of the sample by means of tensile glue, after the adhesive was fully cured, connect the tensile test column to the adhesion tester and perform the tensile test.

### 2.5. Corrosion Resistance Test

A Princeton P4000 potentiostat from Germany was used to measure the open circuit potential (OCP), potentiodynamic polarization, and electrochemical impedance spectroscopy (EIS). A conventional three-electrode electrochemical cell was used for the electrochemical tests. The exposed area of the sample for electrochemical testing is 1 cm^2^, and the corrosive medium was a 3.5 wt. % NaCl solution. Furthermore, a constant temperature water bath at 30 ± 1 °C was used for the tests. The sample was immersed in solution to reach a steady state and then tested at open circuit potential.

For the potentiodynamic polarization tests, polarization was set at a scan rate of 0.333 mV/s. cathodic polarization ranged from −0.3 V to E_oc_, and anodic polarization ranged from E_oc_ to 1.6 V, or a resultant current exceeding 0.01 A/cm^2^. The EIS was tested with a 10 mV sinusoidal perturbation signal, with a frequency range of 10^5^ to 10^−2^ Hz. The EIS data were analyzed and fitted by ZSimpWin. The salt spray test was performed according to the ASTMB 117-03 (5 wt. % NaCl, 35 °C, pH: 6. 5 to 7.2).

## 3. Results

### 3.1. Characterization of MAO Layers Activated by Various Methods

**Figure 2:**
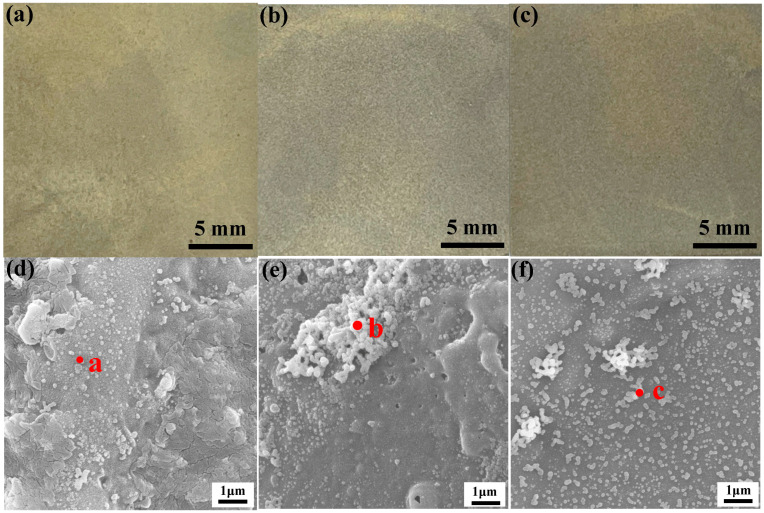
Macroscopic and microscopic morphology of MAO coating activated by different methods: (**a**,**d**) 2 g/L AgNO_3_; (**b**,**e**) 10 g/L AgNO_3_; (**c**,**f**) 10 g/L AgNO_3_+ Ultrasonic.

The morphology of the MAO coatings after being processed with various activation treatments is presented in Figure 2. The surface of the activated samples becomes yellowish green, and no obvious difference in the macroscopic morphology could be observed for various activation methods (Figure 2a–c). The unevenly dispersed nanoparticles could be observed on the surface of the MAO coating at low concentrations of silver nitrate (Figure 2d). The particles could be confirmed as Ag by the EDS analysis of point A in Table 3. For the following deposition process, the Ag particles would play an extremely relevant role, namely both the adsorption and reduction of Ni^2+^ would be taking place on these nucleation sites. For the purpose of confirming whether the increase in active particles had an effect on the performance of Ni-P coatings, the content of silver nitrate was increased to 10 g/L to increase the number of active particles. A difference in surface irregularities could be observed in Figure 2e, and the Ag particles were agglomerated in large quantities. To minimize the agglomeration and clustering of particles, the samples were reduced via ultrasonic assistance. As shown in Figure 2f, the agglomeration of Ag particles was significantly improved. The shock wave formed by the ultrasonic “cavitation phenomena” could destroy the agglomeration of nanoparticles [29,30,31]. A large quantity of Ag particles was uniformly dispersed on the surface of the MAO coating, and the number and distribution of active particles were significantly improved compared to the previous samples.

The EDS analysis of the coatings further revealed the improvements of ultrasonic to the activation process (Table 3). Compared to point B in Figure 2e, the results of point C demonstrate that the agglomeration was significantly improved with the incorporation of ultrasonic.

**Figure 3:**
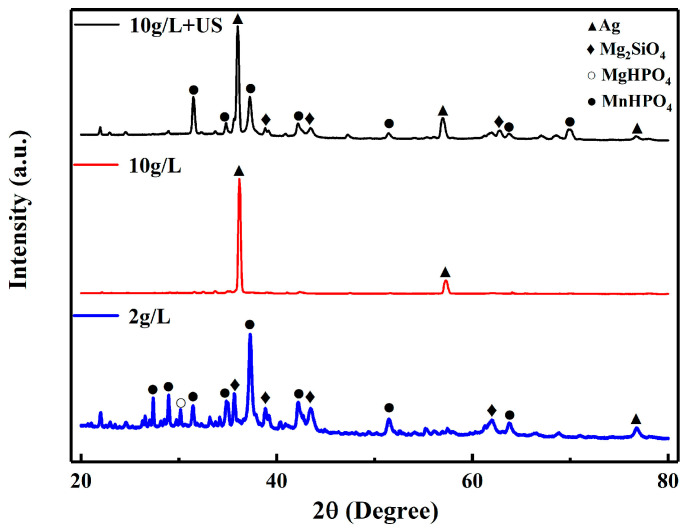
XRD analyses of coatings activated by different methods.

The XRD patterns of the coatings are presented in Figure 3 for different activation methods, respectively. For silver nitrate at a concentration of 10 g/L, almost only Ag peaks could be identified due to the agglomeration of Ag particles, the other components are undetectable due to their relatively low content. For the ultrasonically activated samples, the peak of Ag was definitely identified, as well as other phases also could be detected. Such behavior confirmed the Ag particles were uniformly dispersed.

**Figure 4:**
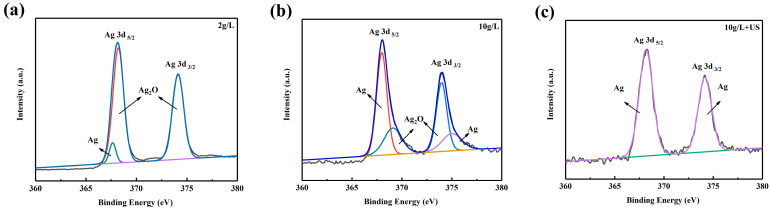
XPS results of coatings activated by different methods: (**a**) 2 g/L AgNO_3_; (**b**) 10 g/L AgNO_3_; (**c**) 10 g/L AgNO_3_+ Ultrasonic.

The XPS results (Figure 4) were also useful to confirm the phenomenon. For the normally activated coating, the appearance of Ag_2_O proved that not all the silver nitrate was reduced to Ag (Figure 4a,b). As a result of sufficient activation, only Ag could be found on the surface of the MAO coating for the ultrasonically activated sample (Figure 4c).

### 3.2. Characterization of Ni-P Coating

#### 3.2.1. Effect of Different Activation Methods on the Deposition Process

To further investigate the effect of different activation methods on the coating during the deposition process, the evolution of the surface of Ni-P coatings was investigated for various deposition times.

**Figure 5:**
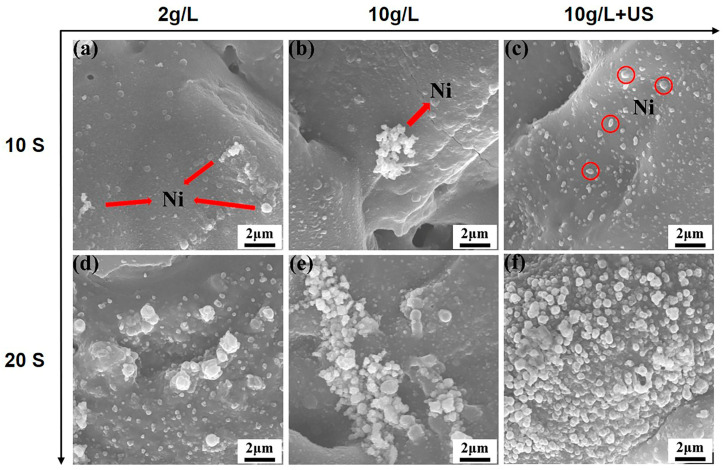
SEM results of the initial deposition process: (**a**,**d**) 2 g/L AgNO_3_; (**b**,**e**) 10 g/L AgNO_3_; (**c**,**f**) 10 g/L AgNO_3_+ Ultrasonic.

Figure 5 presents the SEM results of various activated samples being processed in an alkaline bath for 10–20 s. During the initial 10 s, for the silver nitrate at a concentration of 2 g/L, nickel particles were found to be sparsely distributed on the MAO coating due to the low content of active particles (Figure 5a). As shown in Figure 5b, increasing the concentration of silver nitrate causes significant agglomeration. Figure 5c reveals that a significant improvement of the distribution was observed in the case of ultrasonic activation, and that a considerable quantity of nickel particles are uniformly distributed on the surface of MAO coating. The nickel particles grew significantly and increased in number and size after immersion in the plating bath for 20 s (Figure 5d). For the silver nitrate at a concentration of 10 g/L, the agglomeration of nickel was more pronounced at 20 s (Figure 5e), which may cause less homogeneity for the coating. It should be noticed that homogeneous nickel particles were distributed on the MAO coating at the 20 s for ultrasonically activated samples (Figure 5f), and the grain size was obviously small compared to others, which could be beneficial from a corrosion resistance point of view.

**Figure 6:**
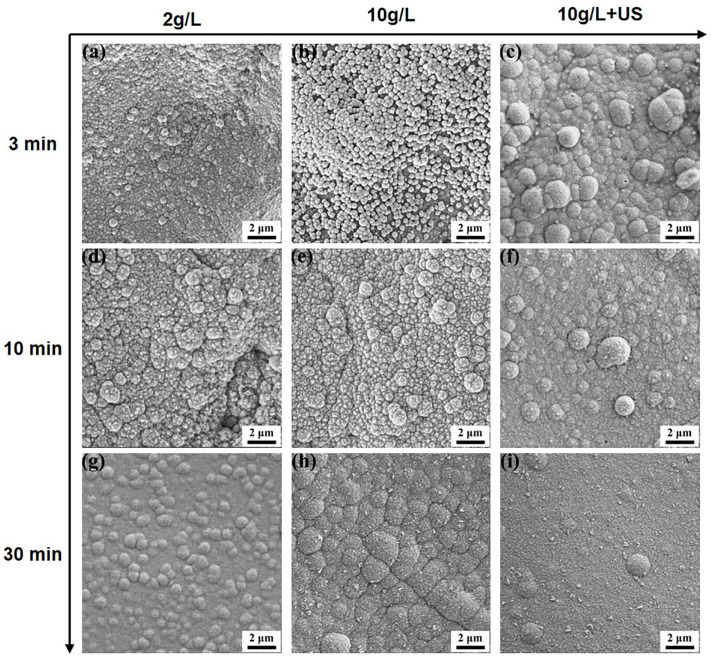
Surface morphology of nickel coatings with different activation methods at different time intervals. (**a**,**d**,**g**) 2 g/L AgNO_3_; (**b**,**e**,**h**) 10 g/L AgNO_3_; (**c**,**f**,**i**) 10 g/L AgNO_3_+ Ultrasonic.

To better understand the depositional behavior due to different activation methods, the surface morphology of the different samples after being immersed in the alkaline bath for 3–30 min was taken (Figure 6). It can be seen that the coatings obtained via different activation methods showed a significant distinction at 3 min (Figure 6a–c). The surface of the sample, which was activated by the silver nitrate at a concentration of 2 g/L, has been completely covered by nickel particles, which were distributed minutely and densely with diameters ranging from 0.1 to 1 μm (Figure 6a). As can be seen from Figure 6b, a large quantity of tiny spherical particles was distributed on the surface of the sample, which activated by the silver nitrate at a concentration of 10 g/L, and some cluster particles have appeared simultaneously while a part of the substrate was still exposed. Moreover, the size of the nickel particles in Figure 6b was significantly larger than that in Figure 6a at 3 min. As shown in Figure 6c, the growth rate of the ultrasonically activated sample was distinctly higher than others. The nickel particles had grown and clustered with each other into larger nodulars, which varied in size from 1 to 2 μm.

At 10 min, the surface of samples in Figure 6d,e were completely covered by a nickel coating. The nodular of both samples was larger than before; however, a high porosity could be observed between the nodulars. The samples in Figure 6f exhibit a flat and compact structure at 10 min. It could be confirmed that the surface morphology of the ultrasonically activated sample was considerably better than others in all cases. As can be seen in Figure 6g–i, the nickel coating was deposited flatly on the surface after 30 min, it could be observed that the apparent pores were still present between the nodular of the samples in Figure 6h, while the coating of the samples in Figure 6g,i had become complete and compact, in which the ultrasonically activated samples were more homogeneous. The SEM results demonstrate that the deposition process of electroless plating was actually affected by the different activation treatments: the increased number of active particles facilitates the adsorption and reduction, which results in a faster coating formation rate.

#### 3.2.2. Cross-Section and AFM Morphology of Different Coatings

**Figure 7:**
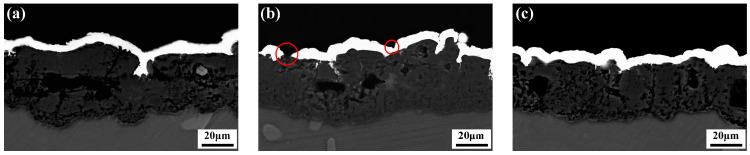
Cross-section observations of coatings after immersion in alkaline bath for 30 min with different activation methods. (**a**) 2 g/L AgNO_3_; (**b**) 10 g/L AgNO_3_; (**c**) 10 g/L AgNO_3_+ Ultrasonic.

Figure 7 illustrates the cross-sectional morphology of different samples after 30 min of immersion in an alkaline bath. For the activation method with silver nitrate at a concentration of 10 g/L, highly pronounced inhomogeneities of the nickel coating were observed, and part of the matrix was still exposed (red circle in Figure 7b), which may result in susceptible to severe corrosion of the MAO layer. Both other activation methods resulted in a complete and compact coating, and the ultrasonically activated sample had a slightly thicker coating than the other samples.

**Figure 8:**
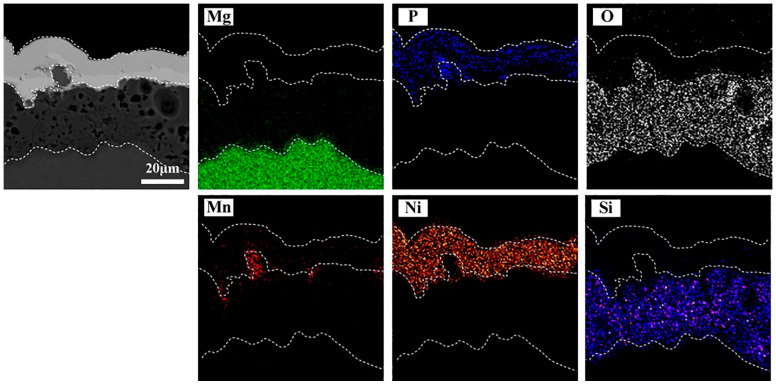
Cross-section observation and EDS analysis of US10-EN composite coating.

Figure 8 shows the cross-section morphology of the US10-EN sample. The coating exhibits a distinct triple-layered structure, with a total thickness measured to be 40–50 μm. There was no phosphorus present inside the MAO layer or the matrix, which is characteristic of compact coating, indicating that the inner layer was not being damaged when the acid bath was performed. The results confirmed that alkaline EN coating plays a major role in protecting the inner layer.

**Figure 9:**
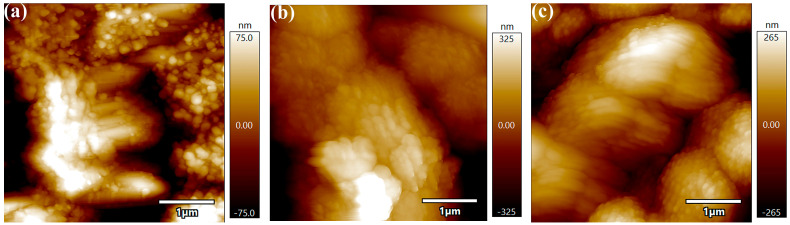
AFM results of coatings after immersion in alkaline bath for 30 min with different activation methods. (**a**) 2 g/L AgNO_3_; (**b**) 10 g/L AgNO_3_; (**c**) 10 g/L AgNO_3_+ Ultrasonic.

The AFM results of different activated samples after being immersed in an alkaline bath for 30 min are shown in Figure 9. Some differences in the surface could be observed for different activation methods. For coatings obtained by activation with silver nitrate at a concentration of 10 g/L, the roughness was slightly higher than others (Figure 9b). This indicates that the agglomeration of active particles causes an increase in the roughness of nickel coating. As could be noticed from Figure 9c, the ultrasonically activated samples exhibited tiny and homogeneous particles and a flat coating.

#### 3.2.3. Conductive Resistance and Adhension Strength Test

**Figure 10:**
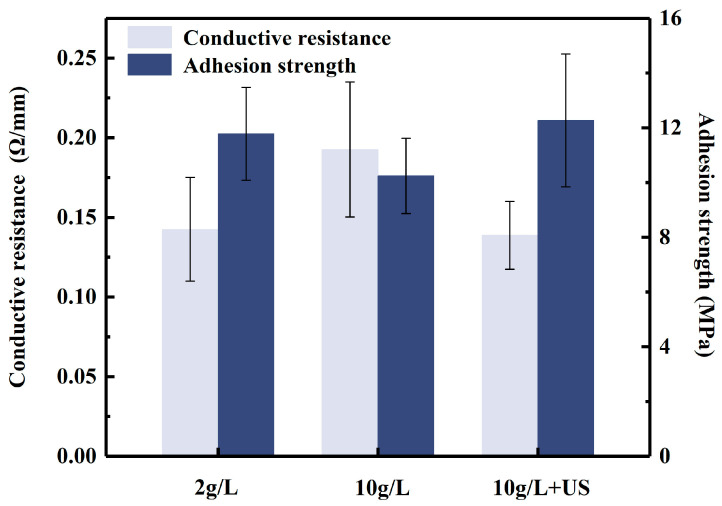
Comparison of conductive resistance and adhesion strength of different coatings.

Figure 10 presents the comparison of the conductive resistance and adhesion strength of the different activated samples immersed in an alkaline bath for 30 min. The ultrasonically activated samples exhibit the lowest conductive resistance and highest adhesion strength. The worst performance was observed for samples activated with a silver nitrate concentration of 10 g/L. As expected, the most probable reason for the phenomena is the inhomogeneity of the coating. Thus, it is likely that the number of active particles plays a major role in the initiation of the deposition process.

#### 3.2.4. XRD Analysis

**Figure 11:**
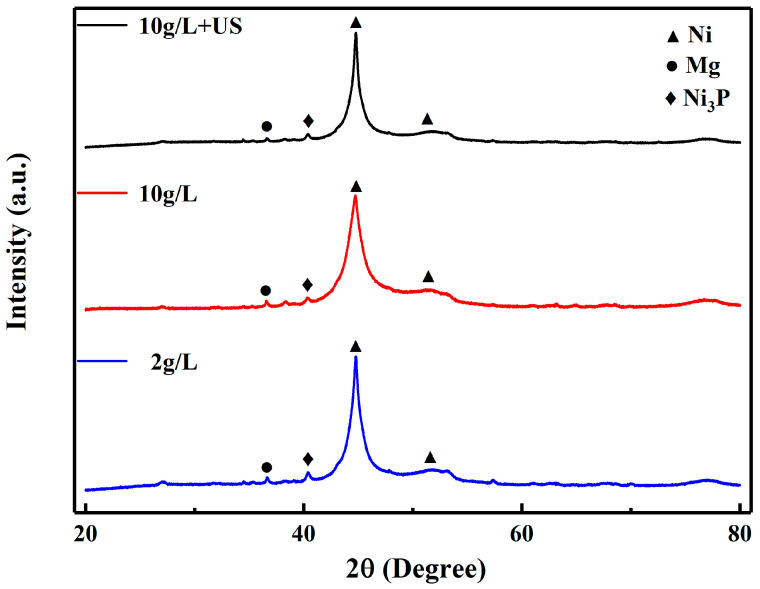
XRD analysis of 30 min alkaline nickel coatings obtained by different activation methods.

The XRD patterns of 30 min alkaline nickel coatings obtained by different activation methods are shown in Figure 11. It can be observed that the phase compositions of the coatings are basically the same, which proves that different activation methods do not affect the composition of the coatings. Combined with the above analysis of the structure of the coatings as well as the bonding and conductive resistance, it can be seen that the MAO coating has been subjected to different activation methods that change the number and distribution of Ag particles, which in turn changes the initial deposition of the coating, thus having an effect on the morphology and properties of the coating, with the exception that this does not change the coating composition.

### 3.3. Corrosion Resistance

#### 3.3.1. Potentiodynamic Polarization Tests

**Figure 12:**
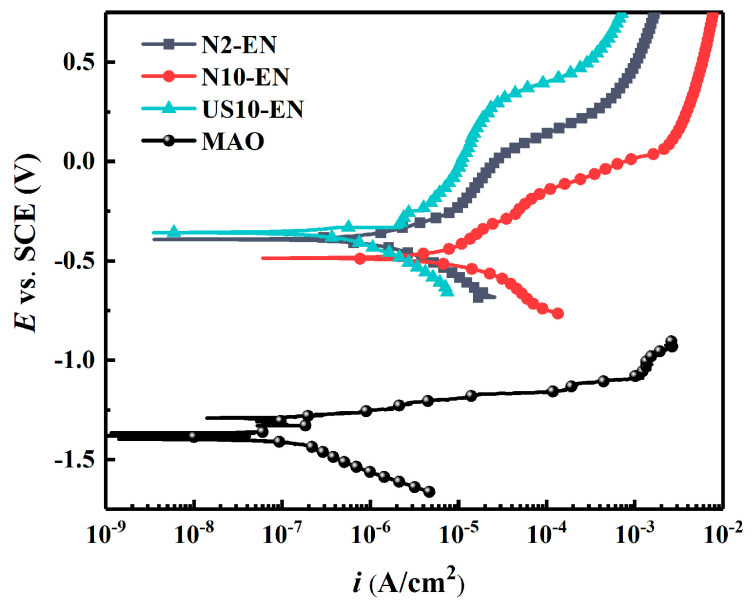
Potentiodynamic polarization curves of MAO coating and composite coatings obtained by different activation methods.

The potentiodynamic polarization curves of N2-EN, N10-EN, and US10-EN samples are shown in Figure 12. The corrosion current density (*i_corr_*), corrosion potential (*E_corr_*), breakdown potential (*E_b_*), and passivation range of the composite coatings could be acquired from the polarization curves. The corrosion current density was determined by Tafel extrapolation [32]. The specific corrosion parameters are summarized in Table 4. Compared to MAO coating, the *E_corr_* of the samples that have been electroless nickel plated was dramatically increased, with the *i_corr_* of the US10-EN samples not differing much from that of the MAO coating. N10-EN has the lowest *E_corr_* of the three samples, and *i_corr_* is obviously higher than the other two samples, indicating a lower corrosion resistance. Compared to the N2-EN sample, the US10-EN sample has a reduction in *i_corr_* by one order of magnitude, and the breakdown potential was increased by about 200 mV, with the range of passivation nearly doubling. As demonstrated through the result, a significant improvement in the corrosion properties was observed for the US10-EN sample.

#### 3.3.2. EIS Tests

**Figure 13:**
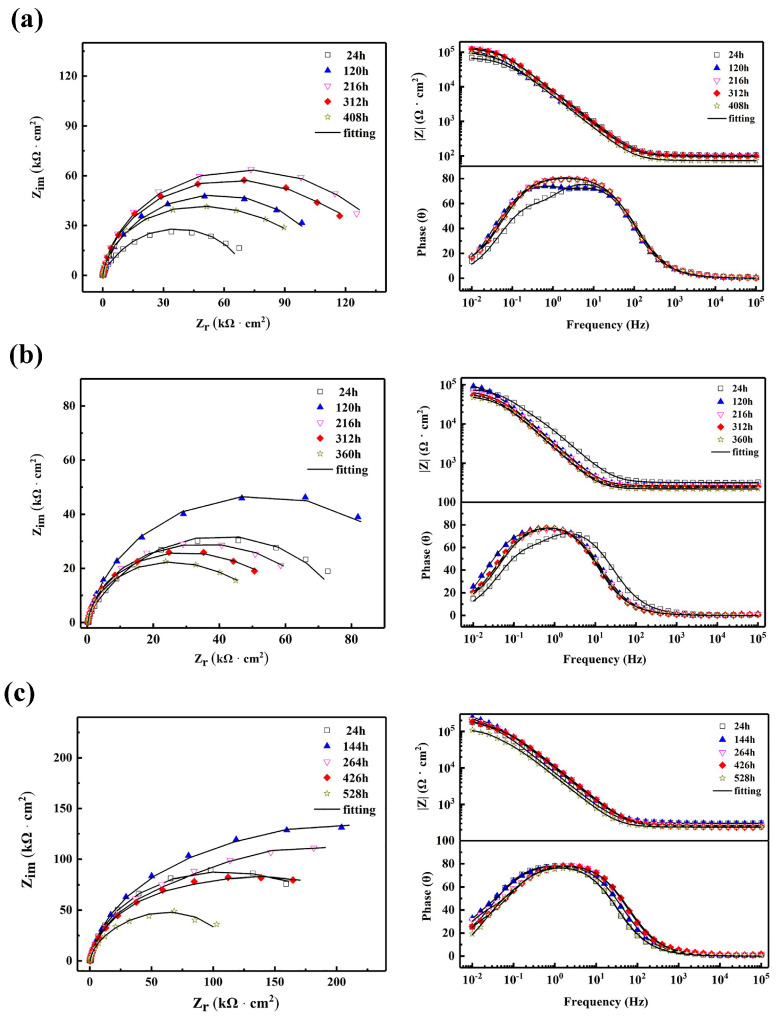
EIS results of different composite coatings immersed in 3.5 wt. % NaCl solution. (**a**) 2 g/L AgNO_3_; (**b**) 10 g/L AgNO_3_; (**c**) 10 g/L AgNO_3_+ Ultrasonic.

Figure 13 presents the EIS results of N2-EN, N10-EN, and US10-EN samples after being immersed for various times. The corrosion resistance can be assessed by the magnitude of the low frequency impedance on the Bode plot [33,34]. The tendency is obvious: the corrosion resistance of all three coatings first increases and then decreases with the increase in the immersion time. Remarkable phenomena should be noted. For example, the diameter of the capacitance loop of US10-EN was consistently larger than that of N2-EN and N10-EN during the immersion process. As a result, the corrosion resistance of the US10-EN sample was significantly greater than that of the other samples, which agrees with the results of the potentiodynamic polarization tests.

**Figure 14:**
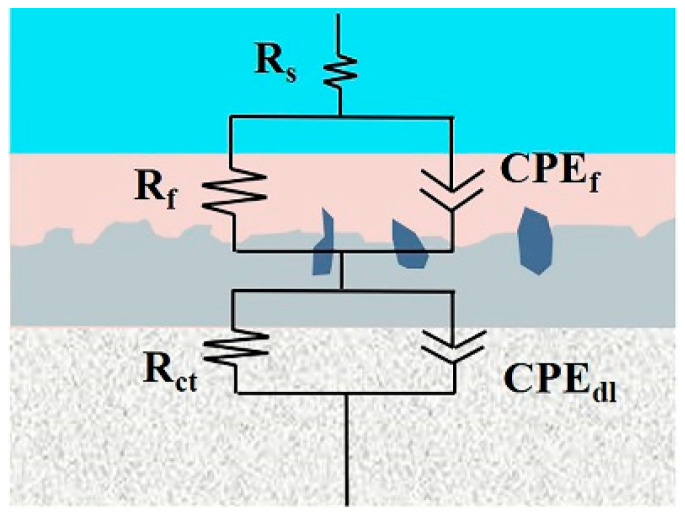
Equivalent circuits used to fit the EIS data.

The equivalent circuit shown in Figure 14 was used to fit the EIS data. Instead of pure capacitance, constant phase elements (CPEs) were used in the equivalent circuit, with the aim of obtaining accurate fitting results [35,36,37]. The *R_s_* represent the solution resistance, *R_ct_* and *CPE_dl_* indicate the charge transfer resistance and the constant phase element of the double layer, while *R_f_* and *CPE_f_* refer to the resistance and constant phase element of the coating [38]. The fitting results are listed in Table 5, Table 6 and Table 7.

**Figure 15:**
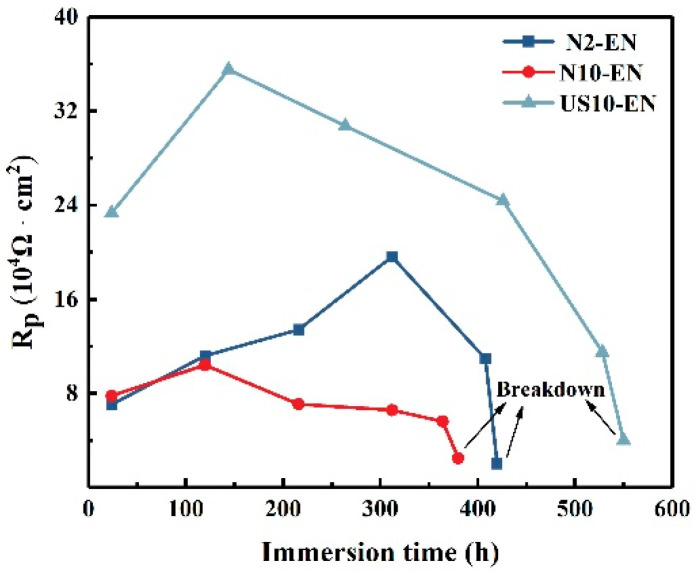
Variation of polarization resistance with immersion time for three composite coatings.

Figure 15 shows the result of the polarization resistance which was plotted according to the calculated results. The polarization resistances of three samples can be calculated by Equation (1) [39]. The polarization resistance were proportional to the corrosion resistance. This indicated that the polarization resistance of all samples tended to increase and then decrease, and that the polarization resistance of the US10-EN sample was remarkably greater compared to that of N2-EN and N10-EN with the extension of the immersion time. The results indicate a significant improvement in the corrosion performance of Ni-P coating in the case of increasing the number of active particles.

(1) $$R_{p}\;=\;R_{f}{\;+\;R}_{ct}$$

#### 3.3.3. Immersion Tests

**Figure 16:**
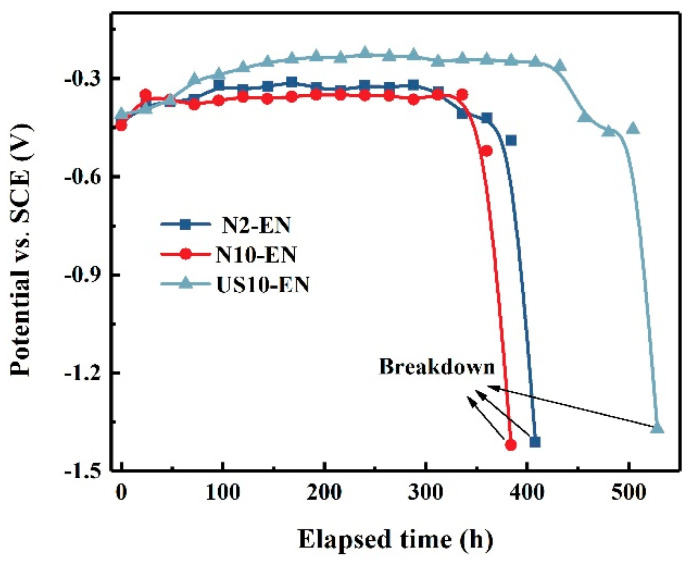
Plot of open circuit potential with time for different samples.

The immersion test gives an indication of the protective properties of different coatings for the substrate. The open circuit potential would drop rapidly as the coating failed due to the attack of Cl^−^. It could be noticed that the open circuit potential of three samples shows a small increase at the beginning and remains at a steady state during the subsequent immersion time (Figure 16). Another result that could be observed is the US10-EN sample reaching a steady state later than the other two samples. It could be deduced from the failure time of coating that the US10-EN coating provides the best corrosion resistance, followed by N2-10 and N10-EN samples, which is attributable to the fact that the coating and the magnesium matrix form a mixed-electrode system. The open circuit potential of the mixed electrode system depends not only on the corrosion potentials of the Ni-P layer, the MAO layer, and the magnesium matrix, but also the surface area ratio of the galvanic coupling of the outer layer and the magnesium substrate [21]. It has been reported that different coatings lead to the different adsorption energy of the chloride ions on the surface of the coating, which results in different corrosion rates [40]. For the early stages of the immersion process, The Cl^−^ ions cannot penetrate the coating and enter the substrate because the outermost electroless nickel layer was complete and compact with low porosity. Therefore, the open circuit potential of the sample was mainly determined by the outermost Ni-P coating. As the immersion time increases, the Cl^−^ gradually infiltrates the interior of the composite coating to the substrate, which certainly contributes to the decrease in the open circuit potential of the sample.

Equations (2)–(4) show the passivation process of Ni-P coatings [41]. During the process of immersion testing, the reaction of nickel with the solution produces Ni^2+^, which dissolves into the solution, contributes to the P enrichment of the coating surface. The reaction between the P-rich layer and the solution formed an H_2_PO_2_^−^ adsorbed layer on the surface, which prevents the dissolution of Ni [42]. Therefore, the passivation was associated with the dissolution of Ni, and there is no doubt that the less porosity the coating has leads to a slower dissolution rate of Ni. In accordance with this conclusion, the reason for US10-EN reaching a steady state last was that the compactness of the coating was the greatest.

(2) $$Ni\to {Ni}^{2+}+2e$$

(3) $$2H+2e\to H_{2}↑$$

(4) $$3P+6H_{2}O\to 3H_{2}{PO}_{2}^{-}+6H^{+}+3e$$

#### 3.3.4. Neutral Salt Spray Tests

**Figure 17:**
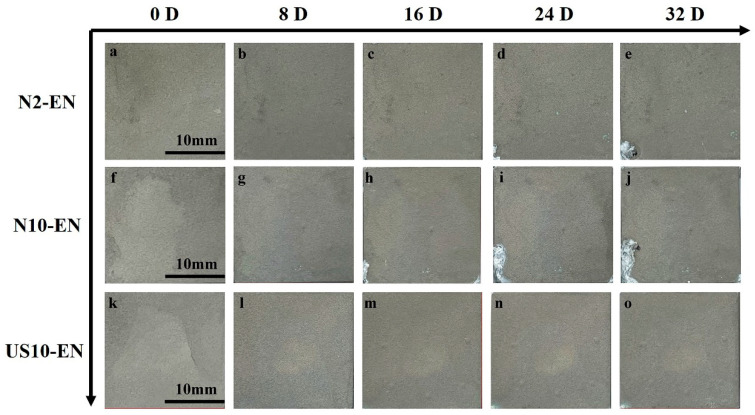
Corrosion morphology of the N2-EN coating (**a**–**e**); N10-EN coating (**f**–**j**) and US10-EN coating (**k**–**o**) with the time of 0 d, 8 d, 16 d, 24 d, and 32 d.

Figure 17 presents the results of the neutral salt spray test for different samples. The samples were not visibly changed during the first 192 h (8 days). Some differences in the surface could be observed for different samples after 384 h (16 days), the corrosion products started to appear on the surface of the N10-EN sample; in addition, the surfaces of the N2-EN and US10-EN remained undamaged. The corrosion area of the N10-EN sample was expanded after 576 h (24 days), and the N2-EN and US10-EN samples were slightly damaged. When the test lasted for 768 h (32 days), the corrosion area of the N10-EN and N2-EN samples was further expanded. In contrast, the US10-EN sample remained a comparatively intact surface, i.e., it was only marginally damaged. The results of the salt spray test corroborate the results of previous experiments that an increase in the number of active particles during activation is beneficial to improve the corrosion resistance of Ni-P coating.

## 4. Influence of the Number and Distribution of Active Particles on the Deposition Process of Nickel Coatings

Typically, compared to coatings obtained from alkaline baths, coatings obtained from acid baths possess a higher phosphorus content and exhibit better corrosion resistance [43,44,45]. However, they become damaged when the MAO coating is directly immersed in the acid bath. Therefore, the sample was immersed in the alkaline bath to achieve an alkaline pre-coating before being immersed in the acid bath. The pre-coating would not only provide protection for the MAO layer, but it also possess catalytic properties. Furthermore, the double Ni layers result in fewer perforated holes, which means superior corrosion resistance compared to the single Ni layer [46]. Therefore, the compactness of the alkaline pre-coating is critical for the corrosion resistance of the composite coating.

The deposition process of electroless nickel coating was similar to the island structure. At the beginning of the electroless plating process, a large quantity of Ni^2+^ moves rapidly to the surface and then is adsorbed on the active particles. These Ni^2+^ were reduced subsequently and functioned as the new catalytic center, and many island structures were formed during the process. With increasing deposition time, the island structure grows continuously, and while in contact and fusing with each other, the final result is a continuous coating on the surface [47].

**Figure 18:**
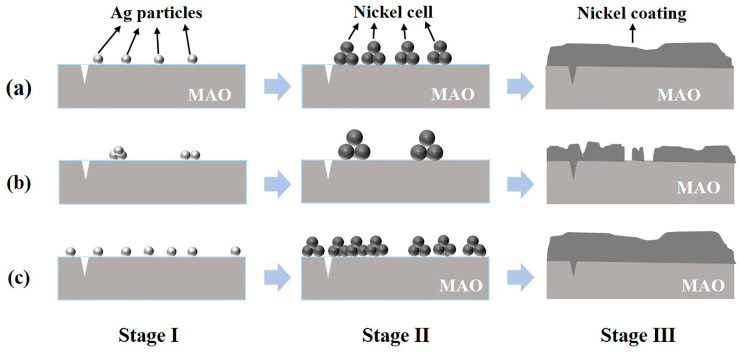
Deposition process of nickel coatings obtained by different activation methods. (**a**) 2 g/L AgNO_3_; (**b**) 10 g/L AgNO_3_; (**c**) 10 g/L AgNO_3_+ Ultrasonic.

Combing the mentioned results, the deposition mechanism of the coating obtained via different activation treatments has been summarized and presented in Figure 18. As compared with Figure 18a, the number of active sites was decreased in Figure 18b due to the agglomeration of Ag particles. Therefore, the growth of nickel particles ceased to be limited by the grains from other sides, and the increased porosity between grains as a consequence of the large size of nickel particles, which implies that the compactness of the coating, will be reduced. Since the plating bath has the same chemical composition, which means that the growth rate of the coating would be equal in parallel or perpendicular directions. The formation of a complete coating will be slow during the same time in case the number of active particles is smaller (Figure 18b). As shown in Figure 18c, the significant increase in the number of active sites was represented by the numerous and uniform dispersion of Ag particles on the surface, which means the number of nickel nuclei were increased during the nucleation stage, restricting the growth of grains and making the pores between the grains become smaller. As a result, more active particles facilitate the formation of a complete coating in a quicker manner during the same time.

## 5. Conclusions

In this paper, we aim to improve the corrosion resistance of nickel coatings by improving the number and distribution of active particles on the surface of the MAO layer. The microstructure, chemical composition, conductive resistance, adhesion, and corrosion resistance of the coatings obtained by different activation methods were discussed. The effect of different numbers of active particles on the deposition process and the properties of electroless plating were investigated

(1) Elevating the silver nitrate content and its reduction via ultrasonic eliminate the agglomeration caused by simply increasing the silver nitrate content during the activation process, which effectively increases the number of active sites on the surface of the MAO layer.
(2) The number and distribution of active particles affect the initial deposition process of nickel particles which affects the compactness of the coating. As the number of active sites decreases, highly pronounced inhomogeneities of the nickel coating are observed. When the number of active sites increases, the nickel particles exhibit a uniform distribution with a smaller size.
(3) Based on the results of conductive resistivity and adhesion tests, the coating obtained from the method with a higher number of active sites results in the best coating conductivity and adhesion.
(4) The results of electrochemical and salt spray tests indicate that the activation method with a higher number of activated particles and uniform distribution provides coatings with better corrosion resistance.

## Figures and Tables

**Table 1:** Activation methods of different samples.

	AgNO_3_ (g/L)	NaBH_4_ (g/L)	Ultrasonic
N2	2	10	×
N10	10	10	×
US10	10	10	√

**Table 2:** Chemical compositions and the experimental conditions.

Process	Formula	Content (g/L)	Experimental Conditions
MAO	Na_2_SiO_3_⋅9H_2_O	20–30	Duty cycle: 30%, Current density: 2.5 A dm^2^, 480 V, 20–30 min
	KF⋅2H_2_O	3–4	
	NaOH	2–3	
MAO-Sealing	MnSO_4_⋅H_2_O	20–40	pH: 3.0−3.2, 55 °C, 5–15 min
	NaNO_3_	1–3	
	NH_4_H_2_PO_4_	20–40	
	EDTA-4Na⋅4H_2_O	2–4	
Sensitization	AgNO_3_	2–10	Room temperature, 30−60 s
Activation	NaBH_4_	10	Room temperature, pH: 13, 30−60 s
	NaOH	2	
Pre-EN	NiSO_4_⋅6H_2_O	20–30	pH: 8.8−9.2, 60 °C, 15–20 min
	NaH_2_PO_2_⋅H_2_O	20–30	
	Na_3_C_6_H_5_O_7_⋅2H_2_O	20–30	
	Na_2_CO_3_	20–30	
	NH_4_HF	5–10	
EN	NiSO_4_⋅6H_2_O	20–30	pH: 5.0−5.5, 80 °C, 80–120 min
	NaH_2_PO_2_⋅H_2_O	20–30	
	C_6_H_8_O_7_	5–10	
	CH_3_CH_2_OONa·3H_2_O	20–30	
	CH_4_N_2_S	1–3 mg	
	(NH_3_⋅H_2_O)	pH adjustment	

**Table 3:** EDS results of chemical composition obtained from selected location in Figure 2.

Element	O	Mg	Si	Ag
Point A	58.74	25.74	14.09	1.43
Point B	56.36	14.95	7.83	20.86
Point C	72.30	14.18	10.68	2.84

**Table 4:** The corrosion parameters of potentiodynamic polarization curves for different composite coatings.

	*E_corr_* (mV)	*i_corr_* (μA/cm^2^)	*E_b_* (mV)	Passive Range (mV)
N2-EN	−394 ± 23	2.04 ± 0.11	302 ± 17	59 ± 28
N10-EN	−485 ± 17	17.1 ± 0.92	154 ± 21	−157 ± 31
US10-EN	−357 ± 19	0.62 ± 0.07	577 ± 24	343 ± 38
MAO	−1402 ± 28	0.37 ± 0.13		

**Table 5:** Fitting results of EIS data for N2-EN samples.

Time (h)	*CPE*_f_ (10^−5^ Ω^−1^ s^−n^ cm^−2^)	*n*_f_	*R*_f_ (10^4^ Ω cm^2^)	*CPE*_dl_ (10^−5^ Ω^−1^ s^−n^ cm^−2^)	*n*_dl_	*R*_ct_ (10^4^ Ω cm^2^)
24	3.77 ± 0.12	0.87 ± 0.03	6.73 ± 2.74	5.66 ± 1.80	1 ± 0.00	0.314 ± 0.048
120	9.78 ± 1.05	0.96 ± 0.09	0.0523 ± 0.0025	3.56 ± 1.01	0.911 ± 0.023	11.1 ± 1.287
216	15.0 ± 2.43	0.93 ± 0.15	0.0181 ± 0.0036	2.61 ± 0.72	0.909 ± 0.129	13.4 ± 2.89
312	50.3 ± 4.62	0.79 ± 0.17	7.99 ± 1.83	2.54 ± 0.98	0.937 ± 0.185	11.6 ± 3.62
408	4.07 ± 0.98	0.95 ± 0.06	6.42 ± 1.64	0.17 ± 0.12	0.851 ± 0.076	4.50 ± 1.24

**Table 6:** Fitting results of EIS data for N10-EN samples.

Time (h)	*CPE*_f_ (10^−5^ Ω^−1^ s^−n^ cm^−2^)	*n*_f_	*R*_f_ (10^5^ Ω cm^2^)	*CPE*_dl_ (10^−4^ Ω^−1^ s^−n^ cm^−2^)	*n*_dl_	*R*_ct_ (10^4^ Ω cm^2^)
24	8.49 ± 1.08	1 ± 0.00	0.332 ± 0.059	3.95 ± 0.51	0.89 ± 0.02	7.47 ± 0.92
120	49.9 ± 5.37	0.98 ± 0.05	0.101 ± 0.032	6.24 ± 1.48	0.93 ± 0.04	10.3 ± 2.36
216	36.1 ± 4.72	0.94 ± 0.08	2.51 ± 0.74	7.19 ± 2.56	0.92 ± 0.07	4.57 ± 1.03
312	131 ± 20.76	0.96 ± 0.13	1.55 ± 0.65	7.60 ± 1.87	0.94 ± 0.06	5.02 ± 0.59
360	7.84 ± 1.98	0.94 ± 0.09	4.34 ± 1.26	1.43 ± 0.36	0.96 ± 0.09	1.27 ± 0.39

**Table 7:** Fitting results of EIS data for US10-EN samples.

Time (h)	*CPE*_f_ (10^−5^ Ω^−1^ s^−n^ cm^−2^)	*n*_f_	*R*_f_ (10^4^ Ω cm^2^)	*CPE*_dl_ (10^−5^ Ω^−1^ s^−n^ cm^−2^)	*n*_dl_	*R*_ct_ (10^4^ Ω cm^2^)
24	2.75 ± 0.24	0.92 ± 0.04	16.5 ± 2.62	34.7 ± 5.99	0.97 ± 0.06	6.76 ± 1.26
144	2.66 ± 0.39	0.90 ± 0.03	12.1 ± 1.79	6.78 ± 1.03	0.94 ± 0.04	23.4 ± 3.67
264	2.30 ± 0.37	0.91 ± 0.07	8.53 ± 2.98	5.99 ± 2.58	0.92 ± 0.12	22.2 ± 3.42
426	7.94 ± 1.28	0.91 ± 0.08	14.7 ± 2.03	2.25 ± 1.12	0.91 ± 0.07	9.66 ± 3.58
528	7.94 ± 1.34	0.97 ± 0.07	8.19 ± 1.24	5.11 ± 1.07	0.89 ± 0.07	3.32 ± 0.98

## Data Availability

Not applicable.
